# Climate Change Impacts on Potato Storage

**DOI:** 10.3390/foods13071119

**Published:** 2024-04-07

**Authors:** Shu Zhang, Xiuquan Wang, Pelin Kinay, Quan Dau

**Affiliations:** 1 Canadian Centre for Climate Change and Adaptation, University of Prince Edward Island, Charlottetown, PE C0A 2A0, Canada; 2 School of Climate Change and Adaptation, University of Prince Edward Island, Charlottetown, PE C1A 4P3, Canada

**Keywords:** climate change, potato storage, potato tuber, vegetative period, pre-storage period, post-harvest

## Abstract

In this study, we present a comprehensive literature review of the potential impacts of climate change on potato storage. Potato preservation can help reduce food loss and waste while increasing long-term food security, as potatoes are one of the most important crops worldwide. The review’s results suggest climate change can negatively affect potato storage, especially tuber sprouting and diseases in storage chambers. Lower Sielianinov coefficient values (indicating dry and hot conditions) during the vegetative season of potato growing can lead to earlier sprouting. For instance, a decrease of 0.05 in the Sielianinov coefficient during the growing season results in tubers stored at 3 °C sprouting 25 days earlier and tubers stored at 5 °C experiencing a 15-day reduction in dormancy. This is due to the fact that the dry and hot climate conditions during the vegetation period of potato planting tend to shorten potato tubers’ natural dormancy, which further leads to earlier sprouting during storage. Furthermore, high Sielianinov coefficient values may lead to worse disease situations. The results also suggest that research about the impacts of climate change on potato storage is very limited at the current stage, and further studies are needed to address the key knowledge gaps identified in this study.

## 1. Introduction

Potatoes (*Solanum tuberosum*) have been human food sources for over 6000 years [1], and they are the fourth largest crop in the world, cultivated in more than 100 countries [2]. Although climate change directly impacts potato post-harvest conditions, including energy usage [3], its effects on potato storage have not been extensively studied.

The objective of potato storage operations is to mitigate quality and weight loss rates. However, achieving these goals faces challenges without thorough consideration of the potato tuber conditions prior to storage. Although a review in 1996 explored how modern techniques, such as refrigerated cold storage and chemical treatments, improve storage conditions for root and tuber crops in tropical areas, considering both pre- and post-harvest factors, it did not specifically address potatoes [4]. Similarly, another review in 2022 examined the factors affecting potato tuber greening [5], but it only considered factors during the storage period, neglecting those occurring before storage. A study from the US used potato production in Michigan to demonstrate how climate change could potentially impact storage conditions for commercial agriculture, with a primary focus on temperature [6]. Understanding the interactions between climatic conditions during the potato growth period and tuber behavior during storage is a crucial component in studying the effects of climate change on potato storage, contributing to the understanding of tuber quality and storage longevity [7,8].

Climate change affects potato storage in many ways, yet there are various unknown challenges and knowledge gaps in this area. Therefore, this study aims to review what has been done and where there are gaps in order to provide suggestions for future research. Specifically, this review will collect literature and examine the following questions: (i) How do climate change considerations affect potato storage, biotically or abiotically? And how will this be accomplished? (ii) How has climate change impacted potato storage management conditions and methods? (iii) Will the financial costs also be affected?

## 2. Methods

**Figure 1:**
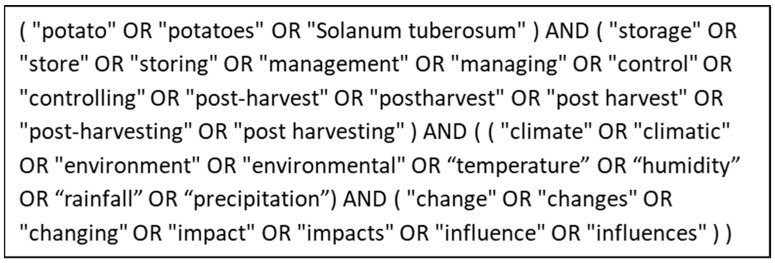
Search strings used to collect relevant literature.

**Figure 2:**
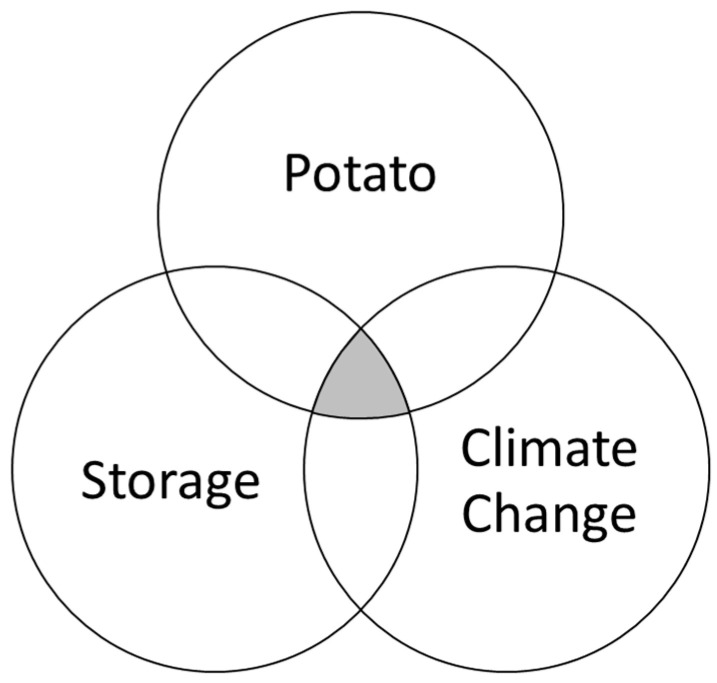
The logic behind the search strings.

The databases used to collect screening literature include PubMed, Scopus, and Web of Science. At the same time, the Wiley Online Library, CINAHL (Cumulative Index to Nursing and Allied Health Literature), and Google Scholar were used for manual searches to find relevant literature, such as referenced studies. Figure 1 shows a range of search strings that were used to derive the resources from the libraries. These string operations were logically manipulated according to the Venn diagram (see Figure 2).

After retrieving results from each database, de-duplication was carried out. The de-duplicated studies were further screened at two levels in two steps: (i) title- and abstract-level screening excludes studies that are unrelated to the research topic; (ii) full-text-level screening includes only studies that completely fulfill the inclusion criteria.

Literature is only included if it meets all three qualifying requirements: (i) the literature addresses at least one research question, (ii) the literature was published between 1 January 2000, and 31 December 2023, and (iii) the literature is in English, with open access to full text. Literature that does not satisfy the eligibility requirements is rejected during the process of searching and collecting, title and abstract screening, and full-text screening. During the manual search stage, the same eligibility criteria are adhered to.

## 3. Results

**Figure 3:**
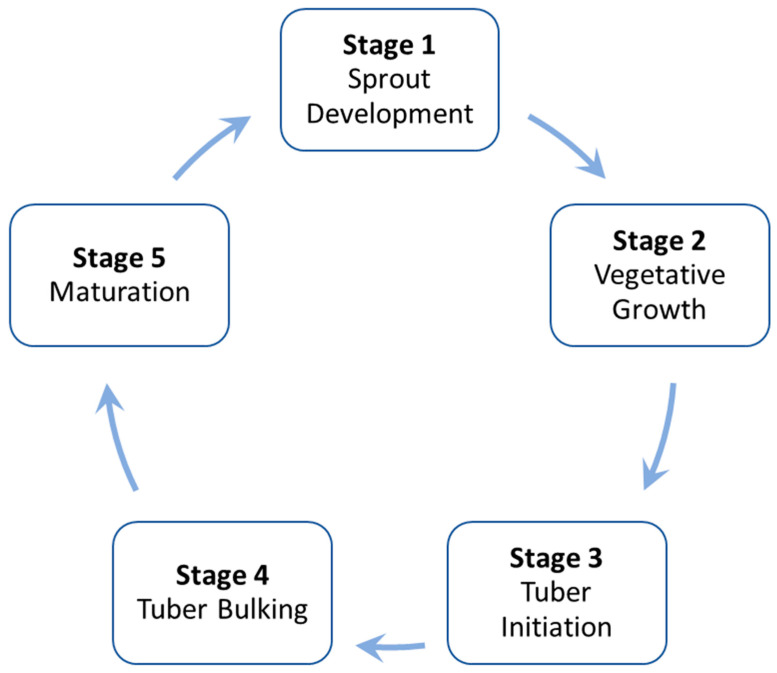
Five stages of potato growth.

A typical potato growth cycle has five stages: Sprout Development (S1), Vegetative Growth (S2), Tuber Initiation (S3), Tuber Bulking (S4), and Maturation (S5) [9,10] (see Figure 3). S1 starts when sprouts develop from the eyes and ends when sprouts reach out of the soil. S2 is when the vegetative parts (including leaves, branches, roots, and stolons) develop, ending with the beginning tuber developing. S3 starts with tubers forming and ends before they enlarge. S4 is when the tubers enlarge by accumulating water, nutrients, and carbohydrates. S5 typically commences as photosynthesis and tuber growth rates decelerate, the vines turn yellow, and eventually wither, causing the leaves to shed. S5 may not be present in the growth cycle of certain long-season varieties within short-growing-season production areas, such as Russet Burbank [10].

In colder and temperate climate zones, potatoes are grown annually. Given the single-yearly crop cycle and the year-round demand for fresh potatoes, potato storage becomes essential. Some of this production must be stored for a longer period, up to 8 to 10 months, to ensure a continuous and stable supply to the market [11,12], maintaining the best tuber characteristics and minimizing losses and wastage. Potato losses, including weight and quality losses, are not avoidable because of the non-stop respiration of potato tubers. Potato quality will only worsen during storage. This loss or quality degradation is combined with potato genome characteristics and environmental factors, so-called “genotype x environment” interactions [12]. The genotype impacts include, but are not limited to, better natural dormancy and improved temperature tolerance.

**Figure 4:**
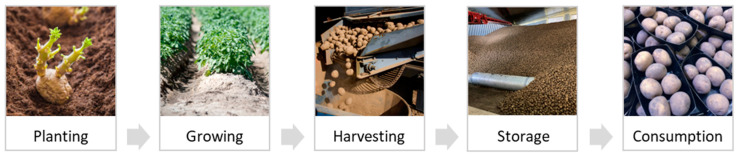
Potato growing is a link in the potato supply chain.

Another factor that needs to be considered is that climate change could directly change the environmental conditions for potato storage. This includes temperature, humidity, gas composition, brightness, etc. It directly affects the abiotic environment and the biotic stresses during potato storage. Moreover, potato quality is dependent on potato variety, pre-storage conditions, storage conditions, and storage duration [13]. The potato supply chain encompasses various stages, including cultivation, harvesting, and storage, extending until the potatoes are purchased from diverse markets (see Figure 4).

Pre-storage conditions include cultivar, growing techniques, type of soil, weather conditions during growth, diseases before harvesting, harvester skills, harvesting and handling equipment, maturity of potatoes at the time of harvesting, moisture stress, nutrient status, and mechanism of tuber damage [13,14].

**Figure 5:**
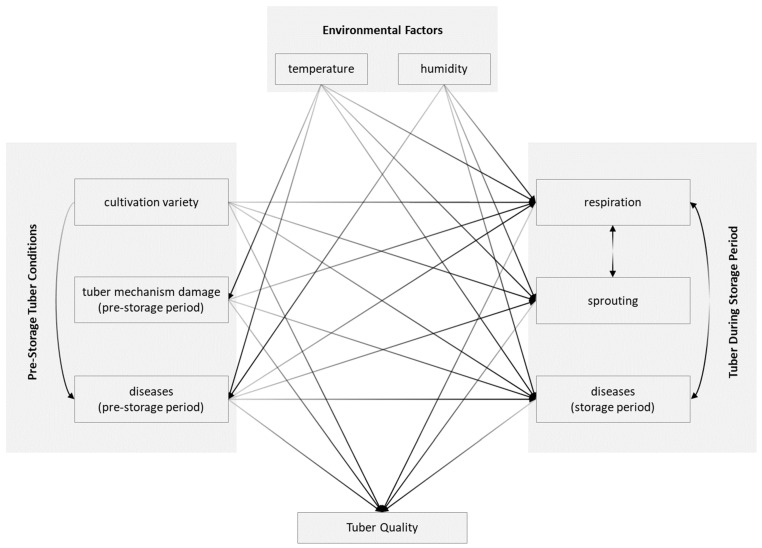
Interactions among environmental factors and potato tubers.

Potato storage faces biotic and abiotic stresses (see Figure 5). Biotic stresses refer to the harmful effects of living organisms, such as diseases. Abiotic stresses are the negative impacts of non-living things, such as temperature, humidity, gas composition, and light. The leading causes of these losses can be categorized into biotic, biochemical, and abiotic factors [14] or physiological, pathological, and insect-related factors [15]. Biotic factors are the living activity of potato tubers or other living things, including sprouting (which leads to weight loss and quality change), pests, and diseases (e.g., blight and soft rot). Weight loss caused by respiration is an example of biochemical change. Temperature (low-temperature sweetening), relative humidity (e.g., pressure flattening), gas composition (black heart), light (greening), and mechanical (skinned tubers) are considered abiotic stresses [14,16]. Changes in the tuber’s chemical composition and physical properties, including moisture loss, decay, color change, and physiological breakdown, are common deteriorations [13]. To achieve the best potato storage, the metabolism of potato tubers and the phenomenon of tuber quality deterioration should be kept at a relatively low level. The tuber’s metabolism includes respiration and sprouting. The quality deterioration phenomenon includes cold-induced sweetening, weight loss, and nutrition loss. The storage strategy of potato tubers for year-round demand should be considered, even when potato varieties are considered, to minimize natural losses and storage diseases and for a longer natural dormancy for potatoes needing long-term storage [8,17,18].

The respiration rate indicates the amount of CO_2_ per kilogram of potatoes produced in 1 h ($g\cdot {kg}^{-1}\cdot h^{-1}$) because of their respiration, indicating the tuber respiration level, which can be used as a disease infection indicator [19]. The higher the respiration rate, the higher the tuber respiration level, and the higher the mass loss. Factors affecting respiration rate include temperature, atmospheric composition, physical injuries, and diseases. Pre-harvest factors like water and heat stresses, shortages or excesses of nutrients, and field-borne diseases can also indirectly affect respiration rates because they may cause physiological or pathological disorders [20].

Potato tuber sprouting is the observable emergence and growth of shoots from the eyes after a period of dormancy. A potato tuber has a natural dormancy period, a transient and reversible stage when visible growth, particularly the emergence of shoots from the eyes, is temporarily suspended. This dormancy phase is vital for the tuber’s survival, and during this period, visible shoot growth is absent. When tubers exit dormancy, the meristematic tissues within the eyes become active, initiating the development of sprouts. Potato tubers do not sprout during the dormancy period after harvesting. However, the dormancy may be broken, and the tubers may start to sprout in extended post-harvest storage. Sprouting will cause changes in nutrients and chemical components, as well as other quality losses. The impact of post-harvest losses in potatoes is shaped by dormancy hormones, where abscisic acid (ABA) and ethylene play roles in inducing dormancy, and an equilibrium between indole-3-acetic acid (IAA) and gibberellic acid (GA) regulates sprouting [21]. Additionally, biotic, entomological, and pathological factors contribute to these losses. Based on the study, dormancy induction requires both ABA and ethylene, while dormancy maintenance requires only ABA. Cytokinins, a class of plant hormones that promote cell division and growth, are the main reason for dormancy break, including cytokinin sensitivity and concentration. IAA is a type of auxin, a plant hormone involved in growth, while GA promotes stem elongation. Changes in endogenous IAA and GA levels are associated with regulating subsequent sprout growth. Sprouting increases respiration, resulting in weight and nutrition losses [22]. The variety, storage temperature, and weather conditions during the vegetation period are the main factors determining when the tubers begin to sprout and the sprout growth rate [23]. Potato sprouting happens after the dormancy break, leading to quality and economic losses [24], and sprouting may happen at any time after the tubers are formed.

Common storage diseases include late blight, wet rot, dry rot, mixed rot, etc. [8]. Soft rot and fusarium dry rot are important post-harvest diseases in potatoes, and disease detection methods have been developed [20]. For instance, Nigeria’s most common rotting agents include *Rhizopus oryzae*, *Fusarium solani*, and *Aspergillus* [25].

### 3.1. Growing Period

The growing period refers to the whole potato-growing stage before harvesting. The climate conditions during this period directly affect the growth of potato plants and tubers and pose continuous impacts on potato tuber storage, affecting potato storability in terms of sprouting date, natural losses, and the amount of diseases during storage [11,26,27,28].

Magdalena and Dariusz [8] used the Sielianinov coefficient (or Sielianinov’s hydrothermal coefficient) to quantitatively describe the weather conditions during the vegetation period for each month from the agroclimatic perspective using the equation *K* = $\frac{P\times 10}{\sum t}$. In this equation, *K* is the Sielianinov coefficient, *P* is the monthly sum of rainfall, and *∑t* is the sum of the average daily air temperature for the given month. The *K* value can impact the sprouting date, natural losses, and the incidence of disease during storage. Three agroclimatic condition categories are used to evaluate the climate conditions based on the *K* value: “dry”, “drought”, and “humid” (see Table 1). However, it’s crucial to note that such classifications are not always fixed. For instance, a four-category classification system used in a 2020 study included drought (0≤K≤0.5), mild-drought (0.6≤K≤1), optimal conditions (1≤K≤1.2), and humid (K>1.2) [29], while in other studies, the classification can be more complicated, such as extremely dry (K≤0.4), very dry (0.4<K≤0.7), dry (0.7<K≤1.0), quite dry (1.0<K≤1.3), optimal (1.3<K≤1.6), quite humid (1.6<K≤2.0), humid (2.0<K≤2.5), very humid (2.5<K≤3.0), and extremely humid (K>3.0) [30,31,32].

#### 3.1.1. Weather Conditions

Hot and dry conditions can shorten the tubers’ natural dormancy, leading to earlier sprouting. Many studies agree with this. A study by Levy and Veilleux [27] argues that high air temperatures and low humidity during the tuber growth period will accelerate the development and reduce the rest period. A study by Czerko and Grudzińska [33] also supports that the Sielianinov coefficient plays a role, and lower coefficient values during the growing season shorten the dormancy period. Specifically, rainfall during the growing season plays an important role in determining the onset of germination. The higher precipitation during this period has the greatest effect on the beginning of sprouting.

Magdalena and Dariusz [8] studied the relationship between the hydrothermal coefficient and the sprouting date at three storage temperatures. They found that, regardless of the storage temperature, the Sielianinov coefficient can estimate the duration and severity of dry conditions from an agroclimatic perspective by indicating the start and end dates of dry conditions. The correlation between the Sielianinov coefficient and tuber sprouting date at three temperatures is presented in Table 2. The highest correlation coefficient observed was 0.972 (*p* < 0.001), while the lowest was 0.674 (*p* < 0.05), corresponding to potato tubers stored at 3 and 8 °C, respectively. Higher Sielianinov coefficient values during the vegetative season of the plant lead to later sprouting. To be specific, the regression equation suggests that a change of 0.05 in the Sielianinov coefficient during the growing season corresponds to a 25-day increase or decrease in dormancy for tubers stored at 3 °C and a 15-day increase or decrease in tubers stored at 5 °C. They used six potato varieties (four medium-early and two medium-late varieties) grown in their experimental station from 2011 to 2013 to examine the impacts of the weather conditions during the vegetation period on sprouting time and weight losses during storage. They stored potato tubers in boxes with molds in storage chambers at 3, 5, or 8 °C. The observation was performed on a 10-day basis. Weight losses were evaluated after 6 months of storage. The losses were categorized into natural losses, storage diseases, and sprouts. The time when 80% of the tubers showed approximately 2 mm shoots was considered the beginning of sprouting. Sprouting length was assessed after complete storage. Note that between 2011 and 2013, the lowest average temperature was 15.8 °C, and the average temperature was 16.1 °C. Similarly, the total rainfall was 355.3 mm in 2012 and 444.2 mm in 2013. The minimum and maximum of the Sielianinov coefficient of the vegetation period were 1.72 (2011), 1.43 (2012), and 1.42 (2013). These values indicated that the conditions for these years were excellent.

Visse-Mansiaux et al. [24] developed predictive models for sprouting forecasts in 2022. In their study, the total daily maximum air temperatures from planting to harvest were considered the second predictor, contributing 70% of the variation in dormancy (the coefficient of determination for validation is 0.70). Their study suggests a negative relationship between the sum of daily maximum temperatures in the air from planting to harvest and potato dormancy. The regression coefficient of 0.02 indicates the change in the dependent variable (potato dormancy) for a one-unit change in the predictor variable (sum of daily maximum temperatures). Temperature during the growing season between germination and harvest greatly influences sprout length during storage. Statistical analysis (*p*-value = 0.02) suggests this factor has a significant effect. At the beginning of the storage period (74 days after harvest), tuber sprouting was low, and there was no significant difference in the length of sprouts between tubers grown at 20 °C (an average length of 0.2 mm) and 15 °C (an average length of 0.0 mm). At 103 days after harvest, tubers grown at 20 °C had a significantly greater shoot length than tubers grown at 15 °C (with an average length of 1.6 mm and 0.3 mm, respectively). Sprout length at 88 and 119 days after harvest was also greater in tubers grown at 20 °C (average of 0.6 and 4.4 mm) compared to 15 °C (average of 0.0 and 2.4 mm). However, it was noted that the differences in the last two observation periods were not statistically significant, even though the p-values were 0.08 and 0.07.

**Figure 6:**
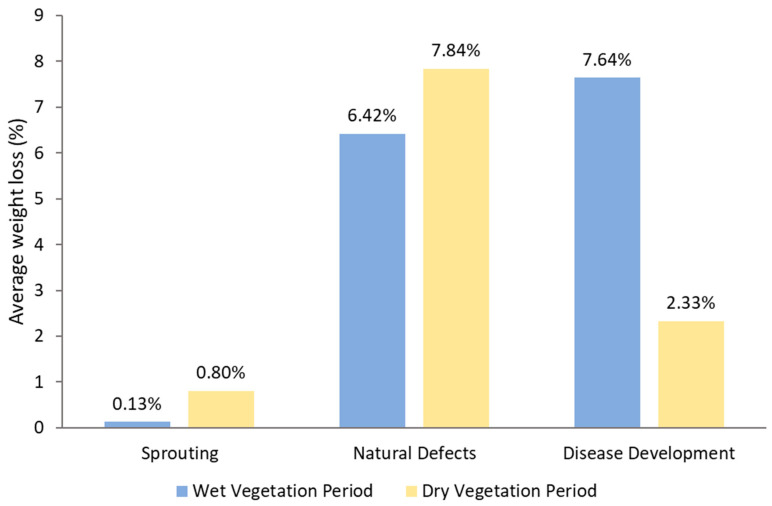
Average weight loss in relation to wet and dry vegetation periods (adapted from [34]).

An experiment by Trawczyński [34] assessed how weather conditions during the growing season affect storage losses. The researcher studied 12 varieties of potatoes grown in 2017 and 2018, then stored in a controlled storage chamber at 5 °C and 90 to 92% humidity for the 2017/2018 and 2018/2019 storage seasons. The varieties include Bohun, Impresja, Lady Rosetta, Lawenda, Madeleine, Magnolia, Lech, Mazur, Otolia, Mieszko, Szyper, and Widawa. His experiment results suggest that both the potato variety and weather conditions significantly impact all types of storage losses (including natural defects, disease development, and sprouting). The wet vegetation period causes more losses in disease development (5.31% more), and dry weather conditions during the vegetation period cause more losses in sprouting and natural loss (0.67 and 1.42% more, respectively) (see Figure 6). Because of dry conditions, more weight is caused by sprouting in tubers grown in dry vegetation, which may be shortened natural dormancy. Out of the 12 varieties, Madeleine had the lowest storage losses, Widawa had the highest for tubers from the dry growing season, and Bohun had the lowest losses, while Szyper had the highest for tubers from the wet growing season. Bohun, Magnolia, and Mazur are the three varieties that have the best storability in the research years.

Another study in China supports that high temperatures can shorten the tuber dormancy period [35]. The researchers planted potatoes in pots (with controlled potting mix and air humidity). After tuber initiation (65 days after planting), they treated the plants with high temperatures (30 °C for the day and 24 °C for the night). Tubers from the heat stress treatment exhibited earlier sprouting than those from the control treatment during post-harvest storage, as evidenced by a statistically significant difference (*p* < 0.01, *t*-test comparing sprouted/total ratios between heat stress and control). Tubers from the heat stress treatment, except for the cultivar Innovator, displayed sprouting by Day 106 of storage, while minimal sprouting occurred in tubers from the control plants. Though cultivar Innovator demonstrated the longest dormancy among all studied cultivars, Innovator tubers (with heat-sprouted treatment) sprouted on Day 106 of storage. In contrast, tubers from the control group remained sprout-free. The conclusion that “potato tubers grown in summer face the challenge of pre-harvest sprouting and shortened post-harvest dormancy because of the sore factor of heat stress” can be drawn. Shortened post-harvest dormancy leaves the storage industry with a shorter time window to transfer the tubers from the field to the warehouse, posing potential increases in financial costs and risks.

Climate conditions during the growing season also affect tuber diseases. For example, a study in Poland in 2021 demonstrates that weight loss due to infection increases with rising air and soil temperatures, as well as with higher Sielianinov coefficient values [32]. Potato late blight is relevant to weather conditions. This disease, caused by the highly variable and adaptive oomycete *Phytophthora infestans* (*Mont.*) de Bary, is perhaps the most well-known and persistently devasting potato disease. This disease has spread since then and now occurs worldwide. The infected tubers are susceptible to soft-rot bacteria, leading to decay during storage. The original strain A1 caused the potato shortage in the 1840s, leading to the deaths and emigration of an estimated three million people [36]. However, A2, a more virulent and aggressive strain, has already taken over and is fast displacing the old strain in many countries [37]. In India, mating type A2 is established in the temperate hills, while A1 dominates in the subtropical plains [38]. Studies reveal that this disease can be forecasted by temperature, rainfall, and relative humidity [38,39]. Bhattacharyya et al. [40] used daily weather data and actual late blight occurrences to predict the conditions. They found that prolonged rainfall and lower temperatures predicted the onset of blight within three weeks, followed by specific humidity and temperature thresholds for more accurate predictions. This model has been effective in Shimla since 1983. Similarly, Prasad and Singh [41] developed a model for the eastern plains based on favorable blight days and canopy density. The researchers in another study [42] also developed a computer-aided forecasting model, JHULSACAST, for western Uttar Pradesh. It considers rainy and non-rainy years and specific conditions related to rainfall, humidity, and temperature to predict the appearance of blight within 10 days. A robust correlation between the hydrothermal coefficient, representing the weather conditions during the growing season, and the impact of Black Scurf Disease (BSD) was evident only in the tubers of the Etiuda variety (no such significant correlation was observed for other cultivars.), with correlation coefficients of *r* = 0.906 and *R*^2^ = 0.8222 [43]. Their study shows that weather conditions do not significantly affect the black spot rate in potato tubers of susceptible varieties, even though variation observation with significant differences was documented.

#### 3.1.2. Soil Characteristics

Soil characteristics can affect potato tuber quality. In Wekesa’s study, significant correlations were observed between soil *pH* and potato tuber dry matter content, as well as phosphorus concentrations, minerals, and vitamin *C* content of potato tubers, suggesting that planting location influenced potato quality [44,45]. The specific gravity, dry matter, reducing sugar, and vitamin *C* content of potato tubers varied significantly (p≤0.05) depending on the place of growth of the plants, the potato varieties, and the storage conditions of the seed potatoes. Interactions between plant location, variety, and seed storage were also significant. According to a study in 2021 [24], the growing location can partly (5.4%) explain the dormancy variability, with a *p*-value less than 0.001. Specifically, the mean dormancy period recorded at the site “Changins” stood at 93 days (*N* = 2138), whereas in the locations “La Frêtaz” (*N* = 576), “Goumoëns” (*N* = 219), “Grangeneuve” (*N* = 330), and “Les Mottes” (*N* = 116), the average dormancy periods were 110, 100, 92, and 88 days, respectively. A study in Uzbekistan shows that the high levels of soil salinity significantly decrease the dry matter and starch content of the tuber but increase the amount of ash and chlorine [46]. The decrease in tuber starch is because chlorine ions have the property of reducing the starch. While high nitrogen fertility can increase potato yield, storage quality is usually negatively impacted [14]. High levels of nitrogen or an excess amount may prolong the maturation process of potato crops, reduce the dry matter content in the tubers, and lead to inferior skin formation. A study investigated the impact of different levels of potassium (2, 4, 8, and 12 meq L^−1^) and sulfur (1, 2, 4, and 6 meq L^−1^) on potato quality and storage behavior [47]. Results show that combining potassium and sulfur can improve tuber quality parameters (such as dry matter and protein content) and shelf life. Additionally, there is a correlation between higher dry matter content (>22%) and increased susceptibility to shattering and bruising in potatoes.

### 3.2. Harvest and Post-Harvest

Weather and soil conditions during harvest are important to potato tuber quality [14]. Soil moisture is crucial to minimize bruising, ideally ranging from 60 to 75%. Tubers’ hydration level affects bruising type and severity. Dehydrated tubers are more prone to blackspot bruising, while hydrated ones tend to experience shatter bruising, with an intermediate hydration level being optimal. Furthermore, soil temperature also plays an important role in potato harvest because it can directly affect tuber pulp temperature. Cold pulp temperatures increase the likelihood of both blackspot and shatter bruising, although the specific type of bruise also depends on the tuber’s hydration level. Cold, well-hydrated tubers are more prone to shatter bruising, while warm, dehydrated tubers are more likely to develop blackspot bruising. The ideal pulp temperature range is between 10 and 16 °C. Warm tubers (warmer than 16 °C) have less bruising than cold ones but are more prone to rot. Similarly, another study also suggests that the total damage increases as temperature decreases [48]. Tuber hydration level also changes as the temperature changes. Managing soil moisture and tuber hydration, along with harvesting at appropriate pulp temperatures, can mitigate bruising risks. After harvesting, tubers are vulnerable to pathogens such as *Pythium* spp. and *Phytophthora ethroseptica*, especially when the temperature of the tuber pulp exceeds 19 °C. Conditions like high temperatures and humidity increase the likelihood of infections. To decrease infection occurrence, it’s important to avoid overwatering late in the season, particularly if temperatures are consistently above 22 °C. Dry soil conditions, on the other hand, can lead to dehydrated tubers, resulting in pressure flattening or internal sprouting during storage. Potatoes harvested early in the season, typically in November–December, exhibit unique characteristics and storage requirements compared to high-quality potatoes harvested later, in February–March. This disparity arises from factors such as the immature skin and higher moisture content of early potatoes. Mechanical damage during harvesting can lead to the formation of black spots on the tubers’ pulp, resulting in quality and economic losses [43].

Pre-conditioning (or curing), a pre-storage treatment, is crucial to limit weight losses and prevent microbial penetration. Pre-conditioning involves subjecting harvested tubers to specific environmental conditions before long-term storage. This process allows the potatoes to heal wounds incurred during harvesting, form a protective layer on the skin, and reduce susceptibility to moisture loss, pathogens, and mechanical damage during storage, benefiting the potatoes’ quality and shelf life. Typically, 15 to 18 °C at 90% relative humidity was found to be the best for curing [49]. Irrespective of susceptibility group, storage duration, and temperature, conditioning tubers for 7 days at 15 °C was the most effective method for mitigating BSD. The reconditioning process yielded the most favorable outcomes with tubers of the Jubilat variety, reducing the BSD rate by approximately 64% in 2013 and around 69% in 2014 [43].

### 3.3. Storage Period

Even though potato storage chambers can generally be considered relatively separate spaces, especially when a manually controlled storage space can be created with a modern validation and cooling system, the storage environment is not isolated from the external climate. Besides genetics, tuber physiological changes and storage losses are mainly affected by storage duration and conditions. Storage condition factors include storage temperature, relative humidity, air circulation, and gas composition, which influence respiration, sprouting, water evaporation of tubers, diseases, alterations in dry matter content, dormancy status, moisture depletion, variations in skin thickness, and extreme temperature damage. Different outlets for stored potatoes require different storage conditions, including seed potatoes, household consumption, industry processing, and raw materials for producing starch or alcohol. Immediate wound healing is essential for all purposes [13,15]. The temperature and relative humidity of the air are the two factors having the most effect in a certain storage chamber [8,50,51,52,53].

#### 3.3.1. Temperature

Temperature is essential in potato storage [54]. Temperature during storage can impact potato tubers in several ways, including bacteria activities, sprouting and sweetening risk, greening occurrence, and the production of acrylamide (a potential carcinogen) [14,15,55,56]. Although low storage temperatures can lead to the degradation of cell membranes [57], they are still commonly used to prolong the storability of potatoes [58,59]. For instance, low-temperature storage can reduce losses due to sprouting, wilting, and diseases [60]. According to the work of Rastovski and Es [51], storage temperature significantly influences potato storability, showing a maximum difference of 5 times in the approximate duration of storage (see Table 3). At a storage temperature of 5 °C, potatoes can be stored for approximately 6 months. At the highest temperature listed, 30 °C, storability reduces to just 1 month. The trend shows an increase in storability as the storage temperature decreases from 30 to 5 °C.

Raju et al. [49] recommend 13 °C for potato storage. However, another study [13] suggests varied temperatures depending on potato use, ranging from as low as 2 °C to as high as 10 °C (see Table 4). Potatoes for fresh consumption can be stored at lower temperatures, whereas those designated for chipping and French frying should be stored at relatively higher temperatures. One reason for this distinction is that potatoes stored at excessively low temperatures tend to produce unacceptably dark fry products [61], due to the accumulation of reducing sugars such as glucose and fructose [60,62].

Q_10_ is a temperature coefficient used to measure the changes in biological or reaction rate due to an increase of 10 °C in temperature [63]. Its mathematical equation is defined as $Q_{10}=\frac{Reaction\;rate\;at\;(t+10\;){}^{\circ}C}{Reaction\;reate\;at\;t{}^{\circ}C}$. Most crops have a Q_10_ range of 2.0 to 2.5 at 5 to 25 °C. The Q_10_ effect will increase microbial activity, resulting in higher rot rates. Increasing temperatures affect post-harvest processing and storage due to the Q_10_ temperature effects [3]. The Q_10_ temperature effect means that a 10 °C temperature increase will cause a double or triple of microbial activity, leading to a higher infection or rotting rate. Therefore, the potato tubers should be kept at a relatively low temperature, if possible. The respiration rate of potatoes at 5 °C is very low (less than mL CO_2_ • kg^−1^ • h^−1^) compared to other vegetables (mushroom, spinach, eggplant, or carrot) [49].

#### 3.3.2. Humidity

Wet conditions can cause rotting in long-term storage. Research indicates that exposure to wet conditions initiates reactions linked to safeguarding against oxidative stress and fortifying defense mechanisms [64]. This, in turn, affects the equilibrium of phytohormones, influencing plant growth and susceptibility to pathogens. Water on the tuber surface could serve as a growth signal, similar to the seed germination process. This can also result in fewer stems and more roots than tubers controlled in dry conditions.

#### 3.3.3. Gas Composition

Gas composition plays an important role in potato storage. For example, low levels of oxygen (O_2_) and/or carbon dioxide (CO_2_) accumulation can cause blackheart disorder [14,65]. According to a study in Europe [66], O_2_, CO_2_, and ethene (ethylene, C_2_H_4_) are the primary active components of the ventilated air in potato storage facilities that affect the storability. Potato tubers are metabolically active and undergo respiration, leading to substantial fluctuations in the concentration of gas components, including water vapor, throughout the storage duration. An atmosphere rich in CO_2_ and with elevated levels of O_2_ can mitigate respiration processing, nutrient loss, and the tendency for browning. Adequate ventilation, typically requiring air exchange rates of 2 to 3 times for around 15 min each, is essential for maintaining proper storage conditions. While there’s ongoing discussion regarding the implementation of CO_2_ sensors for controlling CO_2_ levels, it’s not widely embraced due to the associated high costs of investment and maintenance. Khanbari and Thompson [67] examined the effects of different gas compositions on three crisping potato varieties (Record, Saturna, and Hermes) stored at a temperature of 5 or 10 °C. Their study found that storing all cultivars in 9.4% CO_2_ with 3.6% O_2_ at 5 °C for 25 weeks resulted in nearly complete inhibition of sprouting, minimal weight loss, and preservation of healthy skin.

#### 3.3.4. Light Exposure

Light exposure can affect potato tubers positively and negatively. A study in Norway suggests that exposing seed tubers to continuous light, particularly during the last 3 to 4 months before planting, can inhibit sprout elongation (produce short and sturdy sprouts) and facilitate successful long-term storage [68]. This method offers several benefits, including the potential for earlier (1 to 2 weeks) emergence and accelerated plant growth, earlier harvests, higher early yields, suitability for later cultivars, and reduced incidence of black scurf during storage. The findings indicate that late-maturing potato varieties may particularly benefit from such treatments. However, light exposure may also lead to harmful effects. Glycoalkaloid is a toxic compound that can be found in potato tubers. Tuber glycoalkaloid production is influenced by factors such as mechanical damage, greening, temperature, and light [69]. Although both light and mechanical damage play significant roles in stimulating glycoalkaloid synthesis in potato tubers after harvest, light has been found to have a more pronounced effect. Light is also one of the factors that influence tuber greening, which refers to the phenomenon that the tubers develop a green appearance due to chlorophyll accumulation. Potatoes can undergo greening when exposed to light [15]. This greening occurs because the conversion of amyloplasts into chloroplasts in the tuber’s outer layers is triggered by light exposure.

### 3.4. Impacts on Technologies and Costs

Existing storage methods require large power resources for microclimate systems [16]. Cooling and ventilation are the two most common interventions to control temperature, relative humidity, and gas composition [6,13,20]. Winkler et al. [6] found that there will be more demand for immediate ventilation and/or refrigeration after harvest. Other storage operations include surface drying, lubrication, cooling, sprout inhibition, and controlling storage conditions (temperature, relative humidity, and flow rate). Refrigeration uses around 60 to 70% of the total electricity consumed [70]. However, in winter, potatoes can be stored relatively cheaply because the ambient temperatures are below the storage base temperature. In climatology, agriculture, and energy management, degree days are used to represent the number of degrees and the amount of energy needed to heat up or cool down a building. The higher the number, the more energy is needed. Furthermore, the increasing temperature will increase storage degree days (SDD) [6,71], which can be used as additional energy and financial cost indicators. In their studies, SDDs become 12 °C higher when the potato storage base temperatures are below the ambient temperatures for the first week. According to the study of [6], for the Upper Midwest region in the United States, the percentage difference in potato SDD accumulation demonstrates a mean of 376% from 2020 to 2080 under RCP4.5, with a maximum increase of 28.5% in the early part of the century, a maximum increase of 38.7% in middle of the century, and a maximum increase of 45.5% in late part of the century. At the same time, under RCP8.5, the maximum increase will be 31.1% in the early part of the century, 46.2% in the middle of the century, and 63.2% in the late part of the century, resulting in a 46.8% average SDD increase.

**Figure 7:**
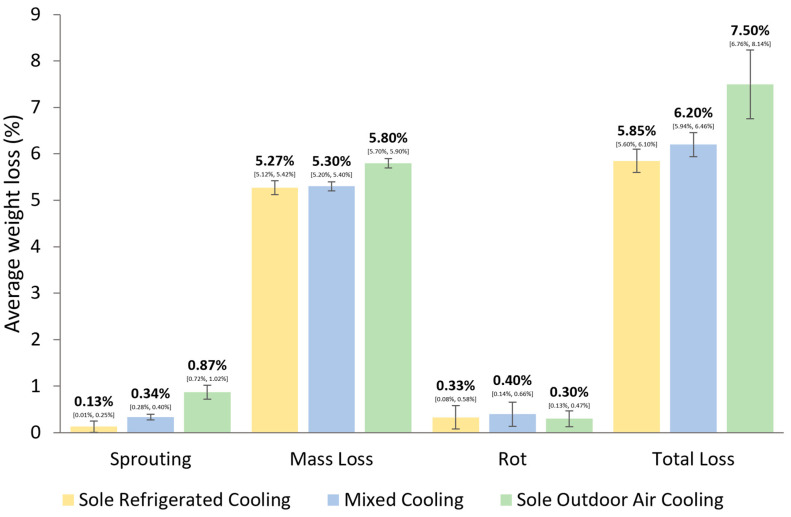
Average weight loss in relation to cooling methods (adapted from [66]).

Nevertheless, the increasing temperature has shortened the winter length, which has resulted in higher energy consumption and a heavier financial burden [6]. In their study, under RCP4.5, the winter length in the Upper Midwest region of the United States will face a maximum loss of 23.7 days in the early part of the century, 27.5 days in the middle of the century, and 30.9 days in the late part of the century, resulting in an average loss of 27.4 days in winter days. Under RCP8.5, the numbers will be 23.6, 31.1, 38.7, and 31.1 days. Different regions are not facing the same level of potato storage challenges. Climate change induces alterations in environmental conditions, impacting traditional methods of potato cooling. Specifically, using fresh air as a cooling agent during a typical storage period (from late summer to early spring) has become more challenging because Europe’s average outdoor temperature has risen in recent years. Therefore, refrigerated cooling is proposed as an alternative to traditional cooling to decrease storage loss and suppress sprouting, involving refrigeration equipment, which increases storage costs in terms of energy and equipment. Different cooling methods can have varying effects on tuber weight losses after storage (see Figure 7) [66]. Cooling with sole outdoor air showed the highest percentage of storage loss (7.50 ± 0.74) and the highest percentage of sprouting loss (0.87 ± 0.15) after storage of 7 months in the 2003/2004 and 2005/2006 storage periods. No significant differences were found in mass loss and rot loss.

Evaporative cooling is used to reduce temperature and increase relative humidity during potato storage in many developing countries to reduce storage costs in short-term storage practices [72]. Using the cooling effect of water evaporation, an evaporative cooling storage system has been used since 2500 BC. Further studies conducted in the 1980s found that the shelf life of different products can be increased by 1.5–6 times [13]. Evaporative cooling costs 90% less than air conditioning and can increase the relative humidity. The projected temperature increase can help the evaporative cooling system maintain thermal comfort [73], and this storage system is being increasingly used [72].

### 3.5. Impacts on Food Security and Sustainability

Climate change also poses threats to food security and sustainability. Feeding the world nutritiously and sustainably faces challenges due to the growing population, and climate change brings more threats to global food systems [74,75,76]. Potatoes, among other food sources, serve as a crucial component within diversified cropping systems. They are crops that contribute to various dimensions of food security, including food availability, access, utilization, and stability, thanks to their current wide cultivation and high demand [75]. They have received strong endorsement from the Food and Agriculture Organization of the United Nations as a food security crop [77]. However, climate change poses significant threats. According to a model, the potato species could potentially face extinction rates of 16 to 22% and experience a range size reduction of more than 50% [74]. Therefore, there is a need to develop cultivars with high tolerance to these stresses [75,78]. As discussed in the previous section, climate change burdens energy consumption, leading to challenges to the economic and environmental sustainability of sustainable food systems [79].

The International Potato Center (CIP) developed its new strategy and corporate plan with consideration of climate change, explicitly focusing on food and nutrition security in identified regions, aiming to strengthen potato development potential, especially in Africa and Asia, as well as the Andes of South America, where the agroecosystems are vulnerable to climate change [77]. Based on IPCC (Inter-governmental Panel on Climate Change) scenarios, many simulation exercises indicate that future potato cropping systems may differ from current practices, implying the need for new varieties to address these changing conditions [80,81]. In this context, two strategies can be explored: (i) maximizing output efficiency by improving input management and optimization, and (ii) minimizing waste by enhancing value chain management, storage, processing, and marketing operations [80].

## 4. Discussion

This review provides an understanding of the impacts of climate change on potato storage. The identified studies were conducted worldwide, in both developing and developed countries. Both quantitative and qualitative data are used.

The literature points out negative associations between climate change and potato storage. Among the examined storage phenomena, sprouting is the most examined in the identified studies regarding the impacts of climate change on potato storage. The shortened natural dormancy due to lower Sielianinov coefficient values is the reason for earlier sprouting during storage. If climate change worsens, more challenges will arise in constraining tuber sprouting during storage. This may lead to decreases in the longevity of storage and increases in the stress of the potato supply before new harvesting. Hence, there is an urgent call for proactive measures to tackle these emerging challenges and maintain the resilience of potato storage systems in the face of ongoing climate variability.

To effectively tackle the challenges posed by climate change, it is essential to consider all aspects of the potato supply chain. For instance, developing and introducing new potato varieties are crucial steps in addressing increasingly severe abiotic and biotic stresses. Additionally, harvest activities must be coordinated with appropriate weather and soil conditions for optimal outcomes.

While the storage chambers can be considered spaces that are separated from the external environment, they still require air exchange to maintain optimal conditions. Despite current efforts to regulate the internal environment, escalating energy costs may pose challenges to both economic and environmental sustainability. Moreover, as external gas composition evolves over time, the limitations of existing ventilation systems may become insufficient. Thus, there is a need to explore additional methods for altering gas composition to ensure the long-term viability of potato storage facilities.

The review only focuses on studies about how climate change impacts potato storage biotic and abiotic conditions, methods, processes, and storage costs. The limitation is that it ignores aspects such as the timing, duration, and demand for potato storage. The need for storage is heavily affected by climate change, which influences the timing of potato planting and harvesting [12]. Additionally, although the possible reason behind the heat-stress-sprouting was explored, other reasons were not discussed in detail.

This review offers valuable insights that can impact stakeholders across the entire potato supply chain, from cultivation to consumption. For instance, growers can leverage the findings to make informed decisions about selecting potato varieties that exhibit greater resilience to prevailing climate conditions in their specific regions, thereby enhancing crop yields and reducing vulnerability to climate-related risks. Harvest activities can optimize the harvesting schedule to avoid adverse weather conditions, minimizing tubers’ mechanical damage and susceptibility to pathogens. Warehouse managers stand to benefit from a deeper understanding of the current storage landscape, which will enable them to implement more effective storage practices and mitigate losses. Ultimately, consumers will enjoy access to higher-quality potatoes, thanks to improved storage and handling practices throughout the supply chain.

Moreover, this review also holds potential benefits for the scientific community. It underscores the urgent need for further research on various aspects of potato storage, particularly in the context of climate change. It highlights the critical oversight that many individuals in the potato storage field may have regarding the continuous influence of pre-storage climate conditions on potato tubers, going beyond mere considerations of storage costs. To gain deeper insights into the complex interactions between climate change and potato storage, future research endeavors may explore questions related to “who, where, and why”.

## 5. Conclusions

The identified studies show that the impacts of climate change on potato storage have attracted more attention; however, this field is still underdeveloped and needs to be examined. Most current studies are devoted to assessing the impacts of climate change on tuber sprouting during storage, with less attention to other phenomena such as diseases.

Despite the limitations of the current studies, the existing evidence points to negative associations between climate change and potato storage. Climate conditions during the potato growing period continuously affect storage after harvesting. The changing climate is increasing pre-conditioning and storage costs. More adaptive cultivation varieties and interventions during the growing season can be considered to better cope with climate change. Further research can be conducted to examine the impact of climate change on potato storage diseases and to enhance public awareness.

## Figures and Tables

**Table 1:** The Sielianinov coefficient and the agroclimatic condition (adapted from [8]).

Sielianinov Coefficient (*K*)	Agroclimatic Condition
0–0.5	Dry
0.6–1.0	Drought
Above 1.0	Humid

**Table 2:** The correlation between the Sielianinov coefficient and tuber sprouting date at three temperatures (adapted from [8]).

Storage Temperature (°C)	Correlation Coefficient	*p*-Value
3	0.972	≤0.001
5	0.826	≤0.01
8	0.674	≤0.05

**Table 3:** Potato storability in relation to storage temperature (adapted from [51]).

Temperature (°C)	Storability (Months)
5	6
10	3–4
15	2–3
20	2–3
25	2
30	1

Note: The tubers were stored in a naturally ventilated store without the use of a sprouting inhibitor (suppressant).

**Table 4:** The ideal storage temperature for potatoes for different uses (adapted from [13]).

Usage	Storage Temperature (°C)
Fresh consumption	2–4
Chipping	4–5
French frying	7–10
Granulation (mashed potatoes)	5–7

## Data Availability

No new data were created or analyzed in this study. Data sharing is not applicable to this article.
